# Metagenome-assembled genomes of papillomaviruses from mallard and northern pintail cloacal swabs

**DOI:** 10.1128/mra.00726-26

**Published:** 2026-08-05

**Authors:** Diego Olivo, Dan Collins, Matthew de Koch, Claire Revekant, Simona Kraberger, Arvind Varsani

**Affiliations:** 1 The Biodesign Center for Fundamental and Applied Microbiomics, School of Life Sciences, Center for Evolution and Medicine, Arizona State University 7864, Tempe, Arizona, USA; 2 Borderlands Research Institute, Sul Ross State University 41054, Alpine, Texas, USA; 3 Bosque del Apache NWR, San Antonio, New Mexico, USA; 4 Structural Biology Research Unit, Department of Integrative, Biomedical Sciences, University of Cape Town 37716, Observatory, Cape Town, South Africa; Katholieke Universiteit Leuven, Leuven, Belgium

**Keywords:** *Anas acuta*, *Anas platyrhynchos*, *Papillomaviridae*

## Abstract

There is little known about papillomavirus diversity in waterfowl. From cloacal swabs of one mallard and three northern pintails sampled in New Mexico (USA), we identified four papillomavirus genomes. These papillomaviruses share >92.7% genome-wide nucleotide pairwise identity with Anas platyrhynchos papillomavirus 3 (AplaPV3) identified from a mallard in Missouri (USA).

## ANNOUNCEMENT

Viruses in the family *Papillomaviridae* have circular, double-stranded DNA genomes ranging in size from 6 to 8 kb (1, 2), and in most cases, they are host-specific, likely due to coevolving with their hosts (3, 4). Most papillomaviruses have been identified from mammals, whereas relatively few have been documented in avian species. To date, complete papillomavirus genomes have only been identified from 15 avian species. In the Anseriformes order, papillomaviruses have only been identified from mallards (*Anas platyrhynchos*) (5–8). Here, we report the genomes of papillomaviruses from a mallard and three northern pintails (*Anas acuta*).

As part of a broader study on viruses associated with waterfowl, cloacal swabs were collected from three northern pintails and one mallard from New Mexico (USA) and stored in 1 mL of UTM Universal Transport Medium (Copan Diagnostics, USA). The samples were collected under the Federal Master Banding Permit #23660 and New Mexico Permit NM #3536. The samples were processed under the Arizona State University’s Institutional Biosafety Committee (#SPROTO202300000004).

Viral DNA was extracted from each sample using the High Pure Viral Nucleic Acid Kit (Roche Diagnostics, USA). Rolling circle amplification (RCA) was used to preferentially amplify circular DNA from extracts with a Phi29 DNA Polymerase Kit (Watchmaker Genomics, USA). The RCA DNA was used to generate sequencing libraries (2 × 150) using the Illumina DNA Prep Kit and sequenced on an Illumina NovaSeq X Plus sequencer at Psomagen Inc. (USA). The paired-end raw reads were trimmed using Trimmomatic v0.39 (9) and *de novo* assembled using MEGAHIT 1.2 (10). Papillomavirus sequences were identified and annotated using Cenote-Taker3 (11) in discovery mode. SDT v1.2 (12) was used to determine pairwise identities. CoverM (13) was used to map raw reads to the genomes to determine read count and depth. All bioinformatic tools were used with default settings.

**Fig 1:**
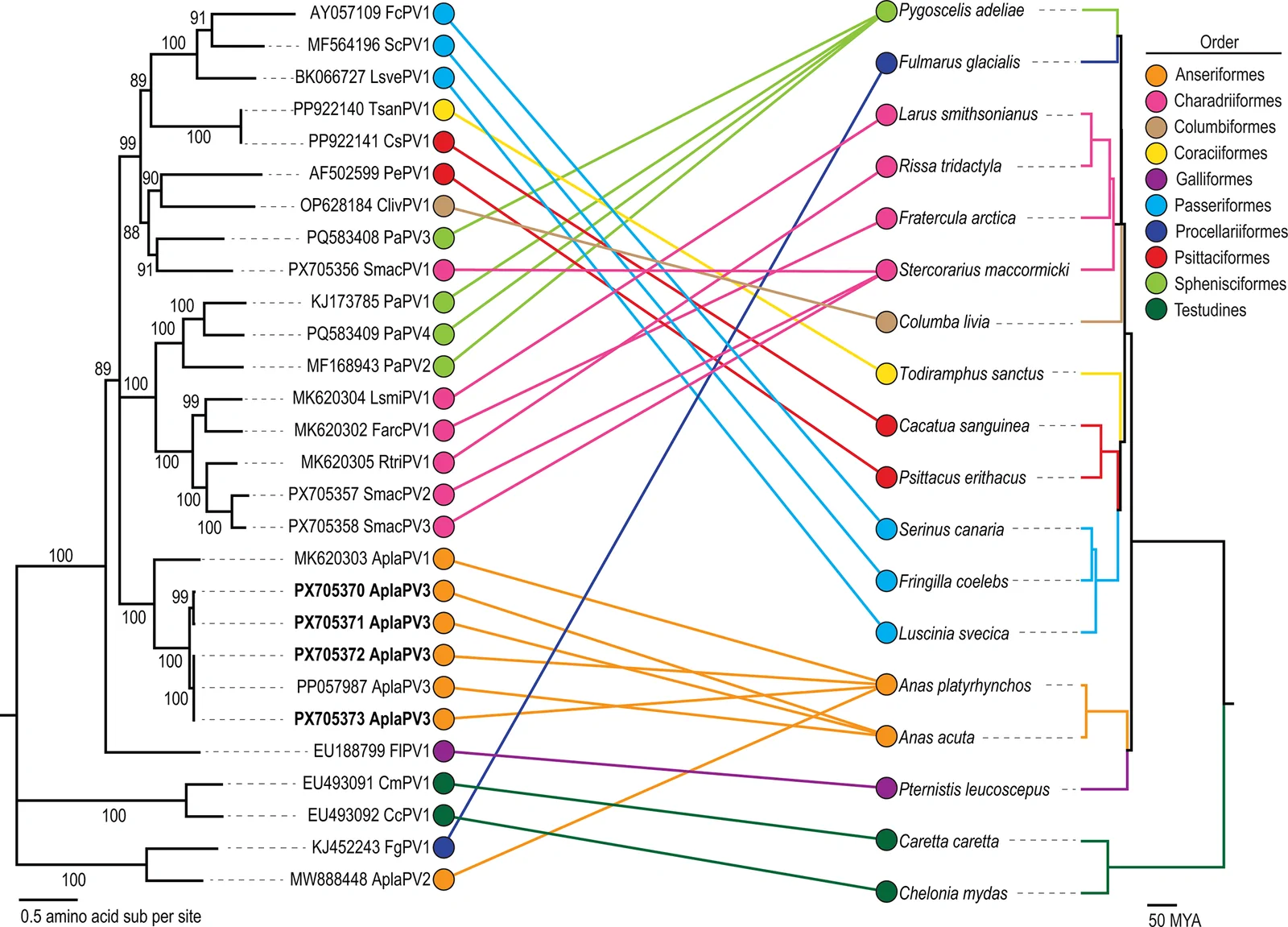
Tanglegram showing the maximum likelihood tree of avian and turtle-associated papillomaviruses (left) and hierarchical host phylogeny (right). For papillomavirus phylogenetic analysis, the avian and related Testudine papillomaviruses were downloaded from the Papillomavirus Episteme (PaVE) database (14, 15). The E1, E2, L1, and L2 genes were extracted and translated from these and aligned using MAFFT v7 (16); the alignments were trimmed using TrimAL v2 (17) with a gap threshold of 0.2, and these trimmed alignments were concatenated. The concatenated alignment was used to infer a maximum likelihood phylogenetic tree using IQTree2 (18) with best-fit amino acid substitution models LG + I + G + F, LG + I + G + F, LG + G + F, and LG + I + G + F for E1, E2, L1, and L2, respectively, determined using ProtTest3 (19). A hierarchical (taxonomy-based) phylogeny of the papillomavirus hosts was constructed using TimeTree v5 (20). The host orders are color-coded, and sequences and hosts from this study are shown in bold.

Four complete waterfowl papillomavirus genomes were identified, three from northern pintails (PX705370–PX705372) and one from a mallard (PX705373) (Table 1), with no visually identifiable disease symptoms. The four papillomavirus genomes share 92.8%–99.8% pairwise identity. These four genomes also share 92.7%–99.9% pairwise identity with Anas platyrhynchos papillomavirus (AplaPV) 3 (PP057987) (8) and thus belong to AplaPV type 3. Although the read depth for AplaPV3 from the mallard (PX705373) (Table 1) is low (5.99×), given that it shares 99.9% genome-wide pairwise identity with one previously determined from a mallard (PX705373) (8), there is high confidence in the sequence assembly. Papillomaviruses are classified based on L1 nucleotide sequence identity, using thresholds of 60% for genus and 70% for species (1, 14, 15). Because AplaPV1 (MK620303) and AplaPV3 share L1 nucleotide sequence identity of 73.9%, they belong to the same species (not yet assigned) and likely represent members of a new papillomavirus genus (Fig. 1).

**TABLE 1:** Summary of papillomavirus genomes identified from the cloacal swabs of *Anas acuta* and *Anas platyrhynchos* with accession numbers, %GC content, number of mapped reads, and mean coverage depth of the mapped reads

Characteristics	Accession			
	PX705370	PX705371	PX705372	PX705373
BioProject	PRJNA1370942	PRJNA1370942	PRJNA1370942	PRJNA1370942
BioSample	SAMN53487133	SAMN53487135	SAMN53487137	SAMN53487141
SRA	SRR36229586	SRR36229584	SRR36229582	SRR36229587
Total # of reads	14,233,947	11,876,889	10,340,070	17,337,301
Source sample	Cloaca swab	Cloaca swab	Cloaca swab	Cloaca swab
Source species	*Anas acuta*	*Anas acuta*	*Anas acuta*	*Anas platyrhynchos*
Papillomavirus type	Anas platyrhynchos papillomavirus 3	Anas platyrhynchos papillomavirus 3	Anas platyrhynchos papillomavirus 3	Anas platyrhynchos papillomavirus 3
Genome length	7,845 nt	7,851 nt	7,839 nt	7,842 nt
Topology, completeness	Circular, complete	Circular, complete	Circular, complete	Circular, complete
GC content	60.3%	60.5%	60.9%	61.0%
Number of mapped reads	35,078	21,533	37,098	301
Mean coverage depth	666.23	409.37	691.56	5.99

The tanglegram (Fig. 1) shows some congruence in the phylogenies of the hosts and papillomaviruses, in particular Anseriformes, Charadriiformes, Passeriformes, and their associated papillomavirus, providing support for host-papillomavirus coevolution coupled with some host switching. This short report expands on the known host range of AplaPV3 to include northern pintails.

## Data Availability

The four papillomavirus genome sequences have been deposited in GenBank under accession numbers PX705370–PX705373. The associated raw sequences have been deposited in SRA under BioProject number PRJNA1370942, BioSample numbers SAMN53487133, SAMN53487135, SAMN53487137, SAMN53487141 and SRA accession numbers SRR36229582, SRR36229584, SRR36229586, and SRR36229587.
